# The Course of Parturition Affects Piglet Condition at Birth and Survival and Growth through the Nursery Phase

**DOI:** 10.3390/ani8050060

**Published:** 2018-04-24

**Authors:** Pieter Langendijk, Marleen Fleuren, Hubèrt van Hees, Theo van Kempen

**Affiliations:** 1 Trouw Nutrition R&D, Stationsstraat 77, 3811 MH Amersfoort, The Netherlands; marleen.fleuren@trouwnutrition.com (M.F.); hubert.van.hees@trouwnutrition.com (H.v.H.); theo.van.kempen@trouwnutrition.com (T.v.K.); 2 Department of Animal Science, North Carolina State University, Raleigh, NC 27695, USA

**Keywords:** parturition, asphyxia, piglet, sow, performance, neonatal

## Abstract

**Simple Summary:**

In this study, data were collected on the condition in which piglets were born, and this was related to their position in the birth order, and to the progress of parturition. The objective of the study was to find out if these observations were related to performance in early life, up to 10 weeks. It appeared that the later the piglets were born in a litter, the higher the risk of being stillborn, and this was aggravated in sows that took a relatively long time to give birth to their litter. In the first few piglets in a litter, risk of stillbirth was only 2%, whereas this increased to 17% in piglets born 13th in the litter or later. Similarly, birth order affected the condition of the liveborn piglets, with blood values such as pH being evident of suboptimal oxygenation in piglets born later. These blood values were predictive of neonatal behaviour such as colostrum intake, but also for neonatal survival and growth during suckling and even to 10 weeks of life. These data are the first in piglets to emphasise the impact of condition at birth on survival and growth until the end of the nursery phase.

**Abstract:**

The aim of this study was to relate the course of parturition to the condition of piglets at birth, based on umbilical cord blood acid-base values, and relate the condition at birth to neonatal survival and performance up to 10 weeks of life. Data were collected from 37 spontaneous unassisted parturitions, and neonatal performance was based on observations of 516 piglets. Stillbirth rate increased from 2% in the first piglets, to 17% in piglets born 13th in the litter or later. This was aggravated in sows with longer than average stage II of parturition. Umbilical cord blood values also reflected the effect of birth order, with pH decreasing and lactate increasing in the course of parturition. Interestingly, sows that had a long expulsion stage of parturition also took longer to give birth to the first four piglets (r = 0.74), suggesting that sows with complicated parturition were already experiencing problems at the start of expulsion of piglets. Piglets with signs of asphyxia, based on umbilical blood lactate higher than 4.46 mmol/L, were slower to start suckling, had a higher risk of neonatal mortality, and had a slower growth rate over the first 10 weeks of life.

## 1. Introduction

In recent publications, stillbirth rate in pigs varies between 5% and 10% [1,2,3,4,5]. Some mortality occurs before the onset of parturition, including mummies and non-fresh stillborns, however, the majority (>75%) of stillborns die intrapartum, due to oxygen insufficiency [6]. Oxygen deprivation during parturition may be caused by stretching and even rupture of the umbilical cord during stage II (expulsion of piglets) of parturition [7], and by the cumulative effect of repeated contractions compressing the placenta and reducing blood flow to the foetus [8]. The latter was simulated in a caesarian section model [9], where the umbilical cord was clamped for 5 to 8 min, resulting in increased lactate and reduced pH in umbilical cord blood compared to unclamped controls. These symptoms are typical for perinatal asphyxia. Several authors have described this condition, and demonstrated that asphyxia is increased in piglets that are born later in the birth order, and in prolonged parturitions [10,11]. Asphyxiated piglets generally show signs of reduced vigour and viability [6], and have a higher risk of neonatal mortality [11]. Previous studies however, have mainly focused on stillbirth and early neonatal behaviour of asphyxiated piglets, and there is a pausity of data on the survival and performance of piglets throughout the lactation period and thereafter. Therefore, the aim of the presented work was to collect data on the survival and performance of piglets through the first 10 weeks of life, and relate them to birth order, and to condition at birth defined by measures of asphyxia. In addition, data are presented on the progress of parturition.

## 2. Materials and Methods

### 2.1. Animals and Measurements

Animal procedures were approved by the ethics committee of Utrecht University, The Netherlands, under approval code 2012.III.05.0411, and were in compliance with EU Directive 2010/63/EU. Data were obtained from 37 multiparous sows (Hypor Libra, Hendrix Genetics, Boxmeer, The Netherlands) with litters of at least 12 piglets. Sows included in these data were allowed to farrow spontaneously without induction, manual assistance or injections with oxytocin. Sows were placed in farrowing crates a week before the expected farrowing date, and received ear vein catheters around four days before farrowing, to allow serial blood sampling, of which the results are presented elsewhere. Briefly, sows were restrained using a snout rope. A 1 × 1.5-mm PVC catheter (Microtube Extrusions, North Rocks, NSW, Australia) was inserted 50 cm into a lateral or intermediate auricular vein. The exterior part of the catheter was secured in a pouch at the back of the neck and taped using Optiplast tape (BSN Medical, Hamburg, Germany).

The sows used for this study were housed at the Swine Research Centre of Trouw Nutrition R&D (Sint-Anthonis, The Netherlands). Sows were housed in individual farrowing crates with feeding trough and drinker, on slats. Farrowing crates were standard commercial type crates, and sows did not have access to nesting material or other substrates. Despite the recognised benefit of nesting materials to sows around parturition, no substrate was provided because it was expected to increase variation between sows in the measurements in this study, due to the variation between sows in the interaction with substrate. For the piglets there was a creep area with heating lights that were on for the first week after farrowing. Temperature in the farrowing room was kept constant at 23–24 °C. Prior to farrowing, sows were fed 2.7–3.0 kg per day of a commercial lactation diet (Diva Optima Lacto, ForFarmers, The Netherlands), depending on the parity. Sows had ad libitum access to water. Once sows had farrowed (d0), feed allowance was reduced to 2.5 kg/d and then increased by 0.5 kg/d each day from d1, until maximum intake was reached. Lights were on from 7:30 a.m. until 10:00 p.m. Piglets had access to a commercial creep feed from one week after birth (Milkiwean Precoce, Trouw Nutrition, The Netherlands).

During farrowing, the time of expulsion of every single piglet was recorded. Immediately at birth of each piglet, the patency of the umbilical cord, meconium staining, and inclusion in membranes was recorded. Of each piglet that was born alive, a blood sample from the umbilical cord was taken immediately after birth by snipping the umbilical cord using scissors, and collecting blood in a heparinised 2.5 mL vial. By the nature of the procedure, the blood sample was a mix of venous and arterial blood (mixed cord sample). Cord samples were analysed by another staff member within 5 min for blood gas values (pH, pCO_2_, lactate) using an iSTAT bed side reader (Abbott, Medini InstruLife, Brugge, Belgium). The piglet was then ear-tagged, weighed, and placed back at the rear of the sow. The procedure took around 2 min. Time to first suckling was recorded. At 24 h after birth of the first piglet, all piglets were weighed again to estimate colostrum intake of individual piglets (CI), using the algorithm developed by Devillers et al. [12], which is based on birth weight and body weight at 24 h, time to first suckling, and the time between the two body weight measurements. Piglets were weighed again at weaning (around 28 days of age), and at the age of 10 weeks.

### 2.2. Statistical Analysis

Statistical analyses were performed using the SAS statistical package (9.3 edition; SAS Institute Inc., Cary, NC, USA). Data on stillbirth and condition of the piglets at birth (measures of asphyxia) are presented in relation to birth order, with birth order pooled for piglet 1 through 3, piglet 4 through 6, etc. (561 total born piglets). Effect of birth order on measures of asphyxia were analysed using PROC GLM in SAS (generalised linear model), with the blood gas values as dependent value, and birth order as independent value. Piglets were then categorised according to their blood lactate value, as a measure of asphyxia, into quartiles with <3.36 mmol/L, 3.36–4.45 mmol/L, 4.45–6.40 mmol/L, and >6.40 mmol/L lactate. In total, data on degree of asphyxia in relation to neonatal performance were available from 516 piglets, and due to space limitation, data on post weaning performance were available from 302 piglets. Percentage of pre-wean mortality, indicators of neonatal viability (time to reach udder, colostrum intake), average daily gain during lactation, and average daily gain from weaning to 10 weeks of life (as dependent variables) were analysed using PROC GLM with the blood lactate class as independent variable. This analysis was repeated using birth weight as a covariate since most of these variables were influenced by birth weight.

## 3. Results

**Figure 1:**
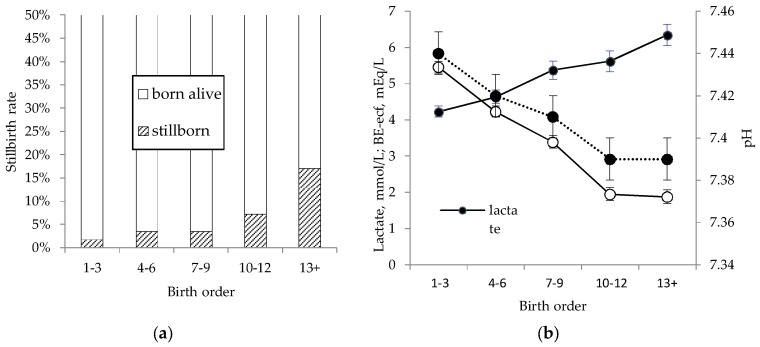
Stillbirth rate (**a**) and blood acid-base values (**b**) in relation to birth order. Data on stillbirth were obtained from 37 litters, and data for blood acid base values from 516 piglets. Mixed umbilical cord blood samples were obtained immediately at birth and analysed within 5 min. BE-ecf: base excess in extra-cellular fluid.

Sows gave birth to 15.2 ± 0.4 piglets on average, of which 14.1 ± 0.3 were born alive, and 1.0 ± 0.5 stillborn. Average stillbirth rate was 6.8% (37 litters, 561 total born). In sows that took shorter than the median (280 min, based on the current 37 sows) to farrow, stillbirth rate was 4% whereas it was 11% in sows that took longer than average to farrow. Stillbirth rate increased with birth order (Figure 1a), from 2% in the first three piglets, to 17% for piglet number 13 and up. The effect of birth order on stillbirth rate was aggravated in sows that took longer to farrow. For the first three piglets the risk of being stillborn was 2%, regardless of whether they were born to a sow with short or long stage II parturition. For piglet number 13 and up, however, the risk of stillbirth was 23% in sows with longer than average stage II parturition, compared to only 9% in sows with shorter than average stage II parturition (*p* < 0.01).

On average, 21% of piglets had a broken umbilical cord at expulsion. This percentage was not related to birth order. Piglets born with a broken umbilical cord had a higher risk of being stillborn (16% vs. 3%, *p* < 0.01) than piglets born with a patent cord.

Indicators for degree of asphyxia in piglets that were born alive reflected an effect of birth order similar to the effect on risk of stillbirth (Figure 1b). As birth order increased, pH in umbilical cord blood decreased from 7.44 ± 0.01 in the first three piglets, to 7.39 ± 0.01 in piglet number 13 and up (*p* < 0.05). Base excess in extra-cellular fluid showed a similar trend, decreasing from 5.45 ± 0.51 mEq/L in the first three piglets to 1.88 ± 0.73 mEq/L in piglet number 13 and up (*p* < 0.05). Lactate showed an opposite trend, increasing from 4.23 ± 0.16 mmol/L in the first piglets to 6.34 ± 0.30 mmol/L in the last piglets (*p* < 0.01). For blood acid base values, there was no interaction between the duration of parturition and the birth order. 

Piglets that were born with a broken umbilical cord had lower blood pH (7.39 ± 0.02 vs. 7.41 ± 0.02, not statistically significant, however biologically significant nonetheless) and higher blood lactate (6.20 ± 0.41 mmol/L vs. 4.91 ± 0.33 mmol/L; *p* < 0.05) compared to piglets with a patent umbilical cord. Meconium staining and whether piglets were born enveloped in membranes did not affect blood acid-base values.

**Figure 2:**
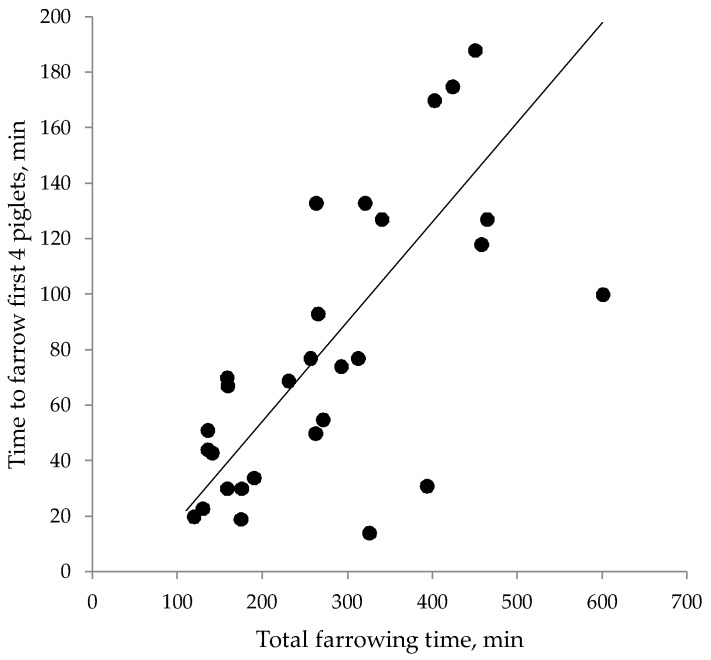
Duration of the entire stage II of parturiton (expulsion of foetuses), in relation to the time taken to farrow the first four piglets. r = 0.74.

Interestingly, the total duration of stage II of the parturition process was strongly related to the time it took to give birth to the first six piglets (r = 0.76; *p* < 0.05), and this relationship was similar for the first four piglets (r = 0.74; *p* < 0.05). In other words, in sows that had a prolonged stage II, signs of a compromised parturition were already evident at the start of the expulsion phase of parturition, in that expulsion of the first four piglets took longer (Figure 2).

Data on neonatal mortality, latency to suck, colostrum intake, etc. are presented in Table 1. Piglets with umbilical cord blood observations were grouped based on their lactate level, as a measure of degree of asphyxia, into four quartiles: those with lactate < 3.36 mmol/L, with lactate from 3.36 to 4.45 mmol/L, from 4.45 to 6.40 mmol/L, and those with lactate > 6.40 mmol/L (Table 1). Neonatal mortality rate (first week of life) was affected by degree of asphyxia, and was 5.5%, 5.4%, 8.5%, and 10.9% (*p* < 0.05) for the four groups, respectively. Piglets with higher lactate level were slower to start suckling, and colostrum intake measured over the first 24 h after birth was lower in these piglets. In addition, the degree of asphyxia was related to average daily gain during the suckling period, resulting in different weaning weights between the four groups. Interestingly, body weight gain was affected by degree of asphyxia until 10 weeks of life, when the period of observation ended, suggesting long lasting effects of asphyxia on performance of young piglets.

It is worth noting that the degree of asphyxia was also related to birth weight, and the average birth weight of piglets decreased from 1.53 ± 0.03 kg in the group with lowest lactate, to 1.25 ± 0.03 kg in the group with highest lactate (*p* < 0.01; Table 1). When birth weight was included as a covariate, the effect of degree of asphyxia on the survivability indicators described above (colostrum intake, daily gain, etc.), was no longer significant. Since birth weight and degree of asphyxia were confounded, it was impossible to separate the effect of birth weight from the influence of asphyxia on neonatal performance.

## 4. Discussion

To our knowledge, this is the first study that reports effects of neonatal asphyxia in a large cohort of piglets, on neonatal survival and performance through the first 10 weeks of life, and the findings emphasise the detrimental effect that oxygen insufficiency during parturition can have on the neonate. This paper also reports that in sows that experience prolonged stage II of parturition, the expulsion of piglets is compromised already from the very start of stage II.

To date, there is poor understanding as to why some sows have a complicated and a prolonged stage II of parturition. This stage is dominated by increased secretion of oxytocin that drives myometrial contractions. Moving or disturbing sows during or shortly prior to parturition reduces contractions and delays expulsion of foetuses, through endogenous opiates inhibiting the release of oxytoxin [13]. Studies by LeCozler et al. [14] and Bories et al. [1] provide some insight into the metabolic changes around parturition. On the day of farrowing, they observed a drop in insulin and urea, and an increase in non esterified fatty acids, probably reflecting fasting or reduced feed intake. Cortisol is generally reported to increase around farrowing [14,15], which may be anxiety related or related to other metabolic changes. LeCozler et al. [14] also reported an increase in P and a drop in Mg on the day of farrowing, probably related to the dephosphorylation of ATP in the uterine muscles to provide energy for myometrial activity. However, none of these studies have provided understanding of complicated parturitions, and exhaustion of energy for myometrial contractions is still generally seen as a cause for prolonged stage II. The data presented here suggest that sows with prolonged stage II are already compromised before the first piglet is born, which means the underlying causes should be looked for in the period before parturition. Some of the conditions leading to compromised stage II may be the frustration of sows not being able to express natural behaviour that is typical of pre-farrowing. Oliviero et al. [16] demonstrated the importance of nest building behaviour in a pen, for oxytocin secretion and the duration of farrowing. Sows confined to crates do not have the opportunity to express this type of behaviour, and presumably also in the period leading up to stage II some of the typical endocrinological changes are affected, which may disturb critical events such as dilation of the cervix. In another study [17], sows with prolonged farrowing did indeed have impaired oxytocin release at the beginning of parturition, and prolonged birth-intervals between the first piglets. It has to be pointed out that in this study no substrate was provided that would have otherwise allowed nest building behaviour.

The data presented in this paper confirm the understanding that prolonged periods of oxygen insufficiency can lead to stillbirth, and neonatal asphyxia [6]. Oxygen insufficiency can occur when the umbilical cord is occluded or ruptured, and this is a major cause of stillbirth [7]. In our study, rupture of the umbilical cord was associated with 16% stillbirth rate as compared to 3% in piglets with a patent cord. Since rupture of the umbilical cord was observed in around 20% of the piglets, one would expect around 3% (16% of 0.20) stillbirth explained by umbilical cord rupture. Stillbirth increased with birth order, whereas the percentage of piglets with a ruptured umbilical cord was consistent across birth order. This means that birth order progressively increased the risk of stillbirth, and this effect was superimposed on the consequences of a ruptured umbilical cord. Interestingly, 57% of stillbirth was associated with a ruptured umbilical cord, whereas Randall [7] reported that 90% of stillborn had a ruptured umbilical cord. This indicates that independent of umbilical cord rupture, the cumulative effect of repeated hypoxic events causes stillbirth and neonatal asphyxia. The effect of birth order is cumulative, and is an increasing build-up of oxygen insufficiency, caused by repeated compression of the placenta and umbilical cord by uterine contractions [8]. Obstruction of blood flow to the foetus induces a temporary drop in heart rate, which in itself is probably harmless and an adaptive mechanism of the foetus to reduce its oxygen consumption. Repeated episodes of obstructed blood flow however, can result in anaerobic metabolism, the cumulative effect of which is an increasing lactate level in foetal blood, and a decreasing foetal blood pH, as buffering capacity is exhausted. This was demonstrated by Van Dijk et al. [9], who clamped the umbilical cord of unborn piglets for 5–8 min during caesarian section, to simulate the cumulative effect of oxygen insufficiency. This explains the profound effect of birth order on stillbirth rate and on neonatal asphyxia. The blood acid-base values in this study reflect the same effect of birth order as in Van Dijk et al. [18], however, the absolute values are different because their data were based on umbilical artery samples as opposed to mix cord samples in the current study. Van Dijk et al. [18] did not monitor effects of asphyxia on neonatal performance. 

The cumulative effect of birth order clearly changed the risk of stillbirth, but also affected the condition of piglets that were born alive, and their neonatal performance. Asphyxiated piglets tend to be slower to start suckling and show less vigour than piglets that are less affected [6], and the present study shows that condition at birth affects survival and performance up to 10 weeks of life. Far more studies on hypoxic-ischemic effects on the neonate have been reported in human neonates. Arteaga et al. [19] reviewed how the oxidative stress induced by hypoxia can cause damage to the brain, other vital organs, and the gastro-intestinal tract in neonates, and the potential therapeutic role of anti-oxidants. It is therefore understandable how in pigs, oxidative damage is reflected in neonatal mortality and reduced growth. The current study demonstrates the long lasting effect that neonatal asphyxia can have on the performance of piglets in early life. Paredes [20] reported that growth retarded piglets had reduced insulin sensitivity and reduced pancreatic amylase activity, compared to non growth retarded litter mates. Piglets from these two categories had similar birth weights. Therefore, post-natal growth retardation may well have been caused by hypoxic conditions at birth, although the authors did not assess blood acid base values.

Interestingly, the degree of asphyxia was confounded with birth weight, and clearly birth weight explained some of the variation in neonatal performance. Although the effect of birth weight and degree of asphyxia could not be separated, they probably both contribute to performance of the neonate. In this respect it is worth noting that birth weight was not related to birth order, and that therefore some of the effects of birth order on hypoxia during parturition are independent of birth weight. The importance of birth weight for neonatal survival was previously discussed by Baxter et al. [21]. Since birth weight is strongly related to size of the placenta [22], it may be speculated that increased birth weight reflects a larger placenta, and that larger piglets therefore are able to cope better with episodes of compromised oxygen supply, due to the increased reoxygenation when perfusion of the placenta is restored between episodes of uterine contractions. If birth weight and the degree of asphyxia are linked, this has implications for increasing litter sizes, since competition in utero increases with litter size and thus compromises foetal growth [23]. 

## 5. Conclusions

In conclusion, this paper demonstrates the cumulative effect of birth order on neonatal asphyxia, and the importance of this condition for performance in early life. This paper also demonstrates that sows with prolonged parturition are compromised from the start of stage II, which has implications for the research into the underlying causes of complicated parturition. The findings underline the importance of providing farrowing conditions to sows that minimise the risk of piglets developing asphyxia, and also the need for management and theraputic measures to recognise the lasting effect of asphyxia.

## Figures and Tables

**Table 1:** Relationship between degree of asphyxia and neonatal performance to 10 weeks of life.

Performance Characteristic	Blood Lactate Concentration (mmol/L)				
	<3.36	3.36–4.45	4.46–6.40	>6.40	*p* Value
*n*	127	127	129	133	
Birth weight (kg)	1.53 ^a^ ± 0.03	1.46 ^ab^ ± 0.03	1.39 ^b^ ± 0.03	1.25 ^c^ ± 0.03	<0.01
Birth to first suckling (min)	34.3 ± 3.2	29.7 ± 3.2	38.8 ± 3.2	39.9 ± 3.3	0.10
Colostrum intake (g) ^1^	463 ^a^ ± 13	441 ^ab^ ± 13	416 ^bc^ ± 13	377 ^c^ ± 13	<0.01
ADG to weaning (g/day)	259 ± 4 ^a^	259 ± 4 ^a^	256 ± 5 ^a^	245 ± 5 ^b^	0.03
Weaning weight (kg)	8.47 ^a^ ± 0.13	8.41 ^a^ ± 0.13	8.13 ^ab^ ± 0.14	7.93 ^b^ ± 0.14	0.02
ADG after weaning (g/day)	721 ^b^ ± 15	710 ^b^ ± 14	717 ^b^ ± 14	664 ^a^ ± 14	0.02

^1^ Colostrum intake was based on increase in body weight between birth and 24 h. ^a,b,c^ Numbers with different superscripts are significantlly different (*p* < 0.05).
